# Effect of citywide enhancement of the chain of survival on good neurologic outcomes after out-of-hospital cardiac arrest from 2008 to 2017

**DOI:** 10.1371/journal.pone.0241804

**Published:** 2020-11-06

**Authors:** Dong Eun Lee, Hyun Wook Ryoo, Sungbae Moon, Jeong Ho Park, Sang Do Shin

**Affiliations:** 1 Department of Emergency Medicine, Kyungpook National University Chilgok Hospital, School of Medicine, Kyungpook National University, Daegu, Korea; 2 Department of Emergency Medicine, Kyungpook National University Hospital, School of Medicine, Kyungpook National University, Daegu, Korea; 3 Department of Emergency Medicine, Seoul National University College of Medicine, Seoul, Korea; Erasmus Medical Center, NETHERLANDS

## Abstract

Improving outcomes after out-of-hospital cardiac arrests (OHCAs) requires an integrated approach by strengthening the chain of survival and emergency care systems. This study aimed to identify the change in outcomes over a decade and effect of citywide intervention on good neurologic outcomes after OHCAs in Daegu. This is a before- and after-intervention study to examine the association between the citywide intervention to improve the chain of survival and outcomes after OHCA. The primary outcome was a good neurologic outcome, defined as a cerebral performance category score of 1 or 2. After dividing into 3 phases according to the citywide intervention, the trends in outcomes after OHCA by primary electrocardiogram rhythm were assessed. Logistic regression analysis was used to analyze the association between the phases and outcomes. Overall, 6203 patients with OHCA were eligible. For 10 years (2008–2017), the rate of survival to discharge and the good neurologic outcomes increased from 2.6% to 8.7% and from 1.5% to 6.6%, respectively. Especially for patients with an initial shockable rhythm, these changes in outcomes were more pronounced (survival to discharge: 23.3% in 2008 to 55.0% in 2017, good neurologic outcomes: 13.3% to 46.0%). Compared with phase 1, the adjusted odds ratio (AOR) and 95% confidence intervals (CI) for good neurologic outcomes was 1.20 (95% CI: 0.78–1.85) for phase 2 and 1.64 (1.09–2.46) for phase 3. For patients with an initial shockable rhythm, the AOR for good neurologic outcomes was 3.76 (1.88–7.52) for phase 2 and 5.51 (2.77–10.98) for phase 3. Citywide improvement was observed in the good neurologic outcomes after OHCAs of medical origin, and the citywide intervention was significantly associated with better outcomes, particularly in those with initial shockable rhythm.

## Introduction

Out-of-hospital cardiac arrest (OHCA) is a continuous public health problem worldwide with low survival rates [1,2]. Improving outcomes after OHCA requires an integrated chain of survival and emergency care systems, including the community elements responding to cardiac arrests, and the healthcare professionals who continue to care for and transport the patients for appropriate treatment [3,4]. Therefore, policy measures appropriate to the characteristics of the region should be implemented to strengthen the chain of survival and improve the OHCA outcomes in the region. Moreover, it is necessary to establish a regional system that monitors the effectiveness of policy measures through investigate the changes in OHCA outcomes and the improvement over time at each part of the chain of survival.

In Daegu Metropolitan City, we have attempted to improve the emergency care system and neurologic outcomes after OHCA. The Daegu Emergency Medical Collaboration Committee, established in 2012, consists of 5 subcommittees: the community intervention division, the emergency medical services (EMS) intervention division, the post-resuscitation hospital care division, and the regional hospital cooperation division. Emergency physicians, local government officials, emergency medical technicians (EMTs), and concerned citizens gathered to improve the emergency care system in Daegu [5]. Each subcommittee conducted intervention efforts according to the field in which they were assigned to strengthen the chain of survival.

In this study, we aimed to identify the citywide outcome trends and the effect of our intervention efforts on good neurologic outcomes after OHCA.

## Materials and methods

### Study setting

The Korean prehospital EMS is operated by the fire department. Daegu Metropolitan City has a population of 2.48 million and an area of 883.54 square kilometers. There is a provincial fire department, 8 local EMS agencies (50 ambulance stations) with a single unified dispatch center, and 25 emergency departments (EDs). One level 1 regional ED and 5 level 2 local EDs provide advanced cardiac life support and post-cardiac arrest care, including target temperature management (TTM), percutaneous coronary intervention (PCI), and extracorporeal membrane oxygenation (ECMO). Nine level 3 emergency rooms and 10 level 4 non-designated urgent facilities provide only advanced cardiac life support. Each prehospital EMS team comprises 2 or 3 EMTs, including a level 1 EMT (similar to EMT-intermediate level in the EMS of the United States) as the top-level ambulance crew, a level 2 EMT (similar to EMT-basic), and a driver. Both scopes of practice are limited to the basic life support level, as has been mentioned in previous studies [6–8]. Since the start of the dual-dispatch system in 2015, two ambulances or one ambulance with one fire engine could be dispatched for OHCA patients. Fire engines were equipped with an automated external defibrillator and firefighters trained for basic life support. The dispatch center selected the type of dispatch, according to the distance proximity and resource availability.

### Citywide interventions

We defined 3 phases of the study period according to the citywide interventions (Table 1). Phase 1 (2008–2011) was the baseline prior to intervention. During phase 2 (2012–2014), community-level intervention and part of prehospital EMS-level intervention were performed. Since 2012, community interventions have been performed to provide appropriate mandatory cardiopulmonary resuscitation (CPR) education. Regional interventions focused on coordinating the competency of regional CPR education by standardizing instructor training and instructor certification. Also, CPR training was provided to the public, especially to those who are required under the Emergency Medical Services Act, such as taxi and bus drivers, police officers, lifeguards, cabin crews, railroad workers, safety personnel, teachers, and child care teachers. In addition, considering the period of CPR knowledge retention, a re-education text message alarm was provided at 6 months, 1 year, and 2 years after CPR training.

**Table 1:** Citywide interventions according to study phase.

Intervention level	Phase 2 (2012–2014)	Phase 3 (2015–2017)
Community	Mandatory CPR and AED training for designated first responders	Mandatory CPR and AED training for designated first responders
		Public access defibrillation program
Prehospital EMS	DA-CPR program	Team CPR program
	Assign a medical director	Dual-dispatch system
Hospital treatment		Standardized guidelines for post-cardiac arrest treatment
		Education programs for emergency physicians, residents and nurses
Link of emergency care system	Establishment and action of Daegu Emergency Medical Collaboration Committee	Regional OHCA Registry
		Public report and feedback to provinces, hospitals and EMTs

*Abbreviations*: CPR, cardiopulmonary resuscitation; OHCA, out-of-hospital cardiac arrest; AED, automated external defibrillator; DA-CPR, dispatcher-assisted cardiopulmonary resuscitation; EMT, emergency medical technician; EMS, emergency medical services.

As part of prehospital EMS-level intervention, the dispatcher-assisted CPR (DA-CPR) program was first implemented in Daegu in 2012. The provincial dispatch center set up the DA-CPR program to detect an OHCA case, instruct how to perform bystander CPR via telephone, and report the process. The program was based on the 2010 American Heart Association (AHA) guidelines [9], which included two simplified key questions to detect OHCA (altered mental status and abnormal breathing) and a structured dialogue to provide high-quality bystander CPR [10]. EMS medical directors have been working in provincial fire departments and 8 local EMS agencies as medical directors since 2012. Each OHCA case was reviewed and scored by the directors for providing feedback to the EMTs.

During phase 3 (2015–2017), implementation of the prospective regional OHCA registry, community- level intervention, prehospital EMS-level intervention, and hospital-level intervention were performed. The community intervention division, in partnership with the Daegu Metropolitan Office of Education and local public health centers, helped to set up the CPR education curriculum in the local schools and public health centers [5]. For prehospital EMS-level intervention, a dual-dispatch system was started to reduce response time and to increase the number of EMTs attending an OHCA [7]. The EMS intervention division, comprising the EMS medical directors, regularly provided a team CPR training program to enable a well-coordinated team approach, with predefined roles for each EMT. Based on the regional OHCA registry [Daegu Emergency Medical Services Registry (DEMSRe)] [7]. We provided feedback on prehospital activities of EMTs and conducted quality-control meetings quarterly. For hospital-level intervention, to increase the professional post-survival treatment rate at the hospital stage, the post-resuscitation hospital care division distributed standardized guidelines for post-cardiac arrest treatment (including TTM, PCI, and ECMO) to level 1 and 2 emergency medical centers in Daegu. Further, the post-resuscitation hospital care division provided educational programs for emergency physicians, residents, and nurses in participating hospitals.

### Study design and population

This is a before- and after-intervention study to examine the association between the citywide intervention and good neurologic outcomes after OHCA in Daegu Metropolitan City. Patients who were aged 18 years or older with OHCA that was presumed to be of medical etiology and who used the EMS system in Daegu Metropolitan City between January 2008 and December 2017, were included. Patients who did not receive resuscitative attempts, who had cardiac arrests witnessed by EMS personnel, or who had cardiac arrests that occurred in a primary care clinic or long-term care facility were excluded from the analysis.

### Data sources and variables

We used the Korean OHCA Registry of the Korea Centers for Disease Control and Prevention, which captures all cases of OHCA in a metropolitan city. Data were retrieved from the EMS run sheets for basic ambulance operational information, the EMS CPR registry, the dispatcher CPR registry, and the hospital OHCA registry for hospital care and outcomes. The medical record review and quality management process has been mentioned in previous studies [6,10].

The following information was collected: demographics including age, sex, past medical history; community factors such as presence of a witness, CPR by a bystander, location of the arrest, and primary electrocardiogram (ECG) rhythm at the scene using the ambulance defibrillator or AED; EMS factors including CPR instruction provided by the dispatcher, activation of the dual-dispatch system, and details of the EMS resuscitation, such as defibrillation, epinephrine, and prehospital advanced airway management by EMTs; time variables such as the response time interval, scene time interval, and transport time interval; and hospital factors such as TTM, PCI, or ECMO.

### Outcome measures

The primary outcome was good neurologic outcome at discharge from the hospital, which was defined as having a cerebral performance category score of 1 or 2. The secondary outcome was survival to hospital discharge.

### Statistical analysis

Demographics and outcomes of the study population during the 3 phases were compared. Descriptive statistics are presented as medians with interquartile ranges (interquartile range: 25th and 75th percentiles), while categorical variables are presented as counts and percentages. The significance of the differences between the three phases was tested (e.g., phase 3 vs. 2, phase 3 vs. 1 and phase 2 vs. 1) using the Kruskal–Wallis test or analysis of variance with post hoc analysis for continuous variables, while the chi-square test was used for categorical variables. All trends were tested by the Cochran–Armitage test.

Associations between the study phase and outcome were assessed using logistic regression analysis. To adjust for potential confounding variables, model 1 was adjusted for demographic factors (age, sex, and comorbidities), and model 2 was adjusted for the demographic factors from model 1 as well as arrest characteristics (witness status, primary ECG rhythm, and location of arrest), and response time interval. For subgroup analysis, we used logistic regression analysis to evaluate the effect of study phase on outcome according to primary ECG rhythm that is defined as a shockable rhythm, such as ventricular fibrillation and pulseless ventricular tachycardia, and non-shockable rhythm, such as asystole and pulseless electrical activity. The results are expressed as adjusted odds ratios (AOR) and 95% confidence intervals (CIs). All statistical analyses were performed with SAS version 9.4 software (SAS Institute Inc., Cary, NC, USA). Based on a 2-sided test, a *P*-value of <0.05 was considered to indicate statistical significance.

### Ethics statement

The study was approved by the Institutional Review Board of Seoul National University Hospital (1103-153-357), which waived the requirement for informed consent.

## Results

### Demographic analysis

**Fig 1:**
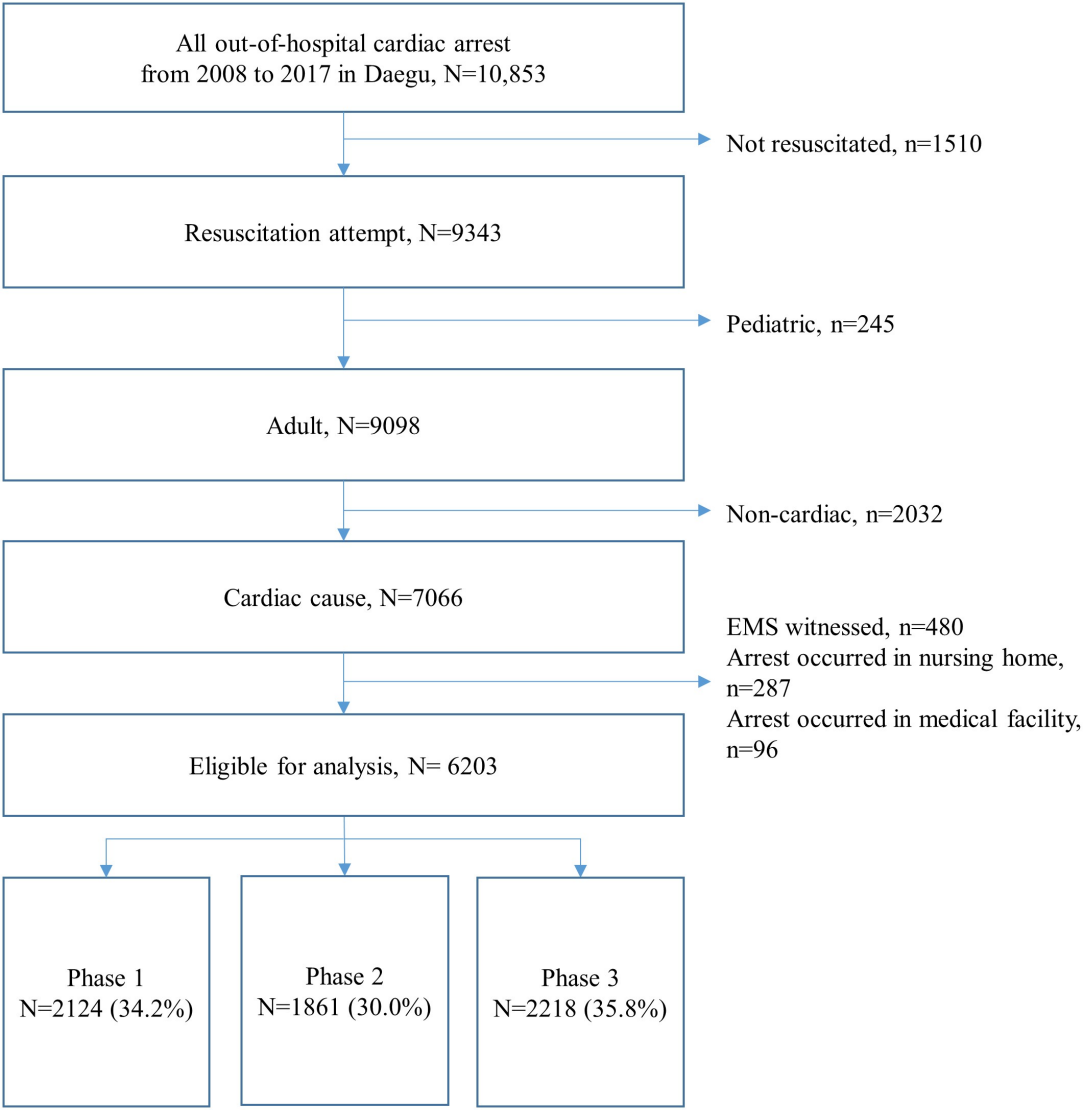
Patient flow diagram.

Among 10,853 EMS-assessed OHCAs in Daegu Metropolitan City, 6203 patients were eligible for our analyses (2124 in phase 1, 1861 in phase 2, and 2218 in phase 3; Fig 1). The demographics are compared among study groups in Table 2. Compared with the phase 1 group, patients in the phase 2 and 3 groups had the following characteristics: older, more comorbidities, more bystander CPR, longer response times and transport times, increased scene time intervals, more advanced airway management, transport to higher levels of ED, more PCI, and more TTM. Patients in the phase 2 and 3 groups had much better outcomes than those in the phase 1 group (all *P* values < 0.001).

**Table 2:** Characteristics of the study population according to the intervention phase.

Variable	All		Phase 1		Phase 2		Phase 3		*P* value^d^	*P* value^e^	*P* value^f^
	N	%	N	%	N	%	N	%			
	6203	100.0	2124	100.0	1861	100.0	2218	100.0			
**Sex**									0.407	0.042	0.213
**Female**	2026	32.7	688	32.4	580	31.2	758	34.2			
**Male**	4177	67.3	1436	67.6	1281	68.8	1460	65.8			
**Age group (years)**									0.153	0.011	<0.001
**≥65**	3642	58.7	1186	55.8	1081	58.1	1375	62.0			
**<65**	2561	41.3	938	44.2	780	41.9	843	38.0			
**Age, median [IQR]**	69 [55–78]		67 [53–76]		69 [56–77]		71 [57–79]		0.001	0.002	<0.001
**Comorbidities**											
**Diabetes mellitus**	1301	21.0	329	15.5	430	23.1	542	24.4	<0.001	0.321	<0.001
**Heart disease**	951	15.3	217	10.2	286	15.4	448	20.2	<0.001	<0.001	<0.001
**Hypertension**	1907	30.7	486	22.9	628	33.7	793	35.8	<0.001	0.180	<0.001
**Stroke**	578	9.3	147	6.9	186	10.0	245	11.0	<0.001	0.277	<0.001
**Location of OHCA**									0.280	<0.001	0.073
**Public place**	1093	17.6	367	17.3	313	16.8	413	18.6			
**Private home**	4425	71.3	1518	71.5	1308	70.3	1599	72.1			
**Other**	685	11.0	239	11.3	240	12.9	206	9.3			
**Primary ECG**									<0.001	<0.001	<0.001
**VF/VT**	633	10.2	107	5.0	261	14.0	265	11.9			
**PEA**	541	8.7	106	5.0	134	7.2	301	13.6			
**Asystole**	5029	81.1	1911	90.0	1466	78.8	1652	74.5			
**Witnessed cardiac arrest**	3016	48.6	1038	48.9	852	45.8	1126	50.8	0.051	0.002	0.212
**Bystander CPR**	927	14.9	98	4.6	218	11.7	611	27.5	<0.001	<0.001	<0.001
**Prehospital defibrillation by public AED**	15	0.2	N/A	N/A	8	0.4	7	0.3		0.548	
**DA-CPR instruction**	1367	22.0	N/A	N/A	409	22.0	958	43.2		<0.001	
**Response time interval (min), median [IQR]**^a^	6 [5–8]		5 [4–7]		6 [5–8]		6 [5–8]		<0.001	0.500	0.006
**Scene time interval (min), median [IQR]**^b^	10 [7–14]		7 [5–10]		9 [7–12]		13 [10–17]		<0.001	<0.001	<0.001
**Transport time interval (min), median [IQR]**^c^	6 [4–9]		5 [3–8]		6 [4–9]		6 [4–10]		<0.001	0.559	<0.001
**Level of EMT**									<0.001	<0.001	<0.001
**1**	4737	76.4	1220	57.4	1445	77.6	2072	93.4			
**2**	1389	22.4	866	40.8	383	20.6	140	6.3			
**3**	77	1.2	38	1.8	33	1.8	6	0.3			
**No. of ambulance crew members**									<0.001	<0.001	<0.001
**1**	150	2.4	20	0.9	110	5.9	20	0.9			
**2**	4031	65.0	1745	82.2	1277	68.6	1009	45.5			
**3**	2022	32.6	359	16.9	474	25.5	1189	53.6			
**Prehospital defibrillation by EMT**	987	15.9	N/A	N/A	458	24.6	529	23.9		0.572	
**Prehospital airway**									<0.001	<0.001	<0.001
**ETI**	359	5.8	19	0.9	32	1.7	308	13.9			
**SGA**	1440	23.2	87	4.1	221	11.9	1132	51.0			
**BVM**	3171	51.1	1382	65.1	1156	62.1	633	28.5			
**PV**	1233	19.9	636	29.9	452	24.3	145	6.5			
**Prehospital epinephrine administration**	78	1.3	N/A	N/A	1	0.1	77	3.5		<0.001	
**Dual dispatch**	1513	24.4	N/A	N/A	N/A	N/A	1513	68.2			
**Level of ED**									<0.001	<0.001	<0.001
**1**	882	14.2	301	14.2	257	13.8	324	14.6			
**2**	4225	68.1	1209	56.9	1274	68.5	1742	78.5			
**3**	766	12.3	405	19.1	237	12.7	124	5.6			
**4**	330	5.3	209	9.8	93	5.0	28	1.3			
**Hospital treatment**											
**PCI**	234	3.8	41	1.9	63	3.4	130	5.9	0.004	<0.001	<0.001
**TTM**	80	1.3	8	0.4	30	1.6	42	1.9	<0.001	0.496	<0.001
**ECMO**	34	0.5	2	0.1	16	0.9	16	0.7	<0.001	0.618	0.001
**Prehospital ROSC**	372	6.0	45	2.1	123	6.6	204	9.2	<0.001	0.002	<0.001
**Survival to discharge**	372	6.0	82	3.9	126	6.8	164	7.4	<0.001	0.440	<0.001
**Good neurologic outcome**	259	4.2	46	2.2	86	4.6	127	5.7	<0.001	0.114	<0.001

*Abbreviations*: IQR, interquartile range; OHCA, out-of-hospital cardiac arrest; ECG, electrocardiogram; CPR, cardiopulmonary resuscitation; AED, automated external defibrillator; DA-CPR, dispatcher-assisted cardiopulmonary resuscitation; EMT, emergency medical technician; ETI, endotracheal intubation; SGA, supraglottic airway; BVM, bag valve mask; PV, passive ventilation; ED, emergency department; PCI, percutaneous coronary intervention; TTM, target temperature management; ECMO, extracorporeal membrane oxygenation; ROSC, return of spontaneous circulation.

^a^Time from call to arrival of the ambulance at the scene.

^b^Time from arrival of the ambulance at the scene to departure from the scene.

^c^Time from departure of the ambulance from the scene to arrival at the ED.

^d^*P* value between phase 1, and phase 2 groups.

^e^*P* value between phase 2, and phase 3 groups.

^f^*P* value between phase 3, and phase 1 groups.

### Trend analysis

**Fig 2:**
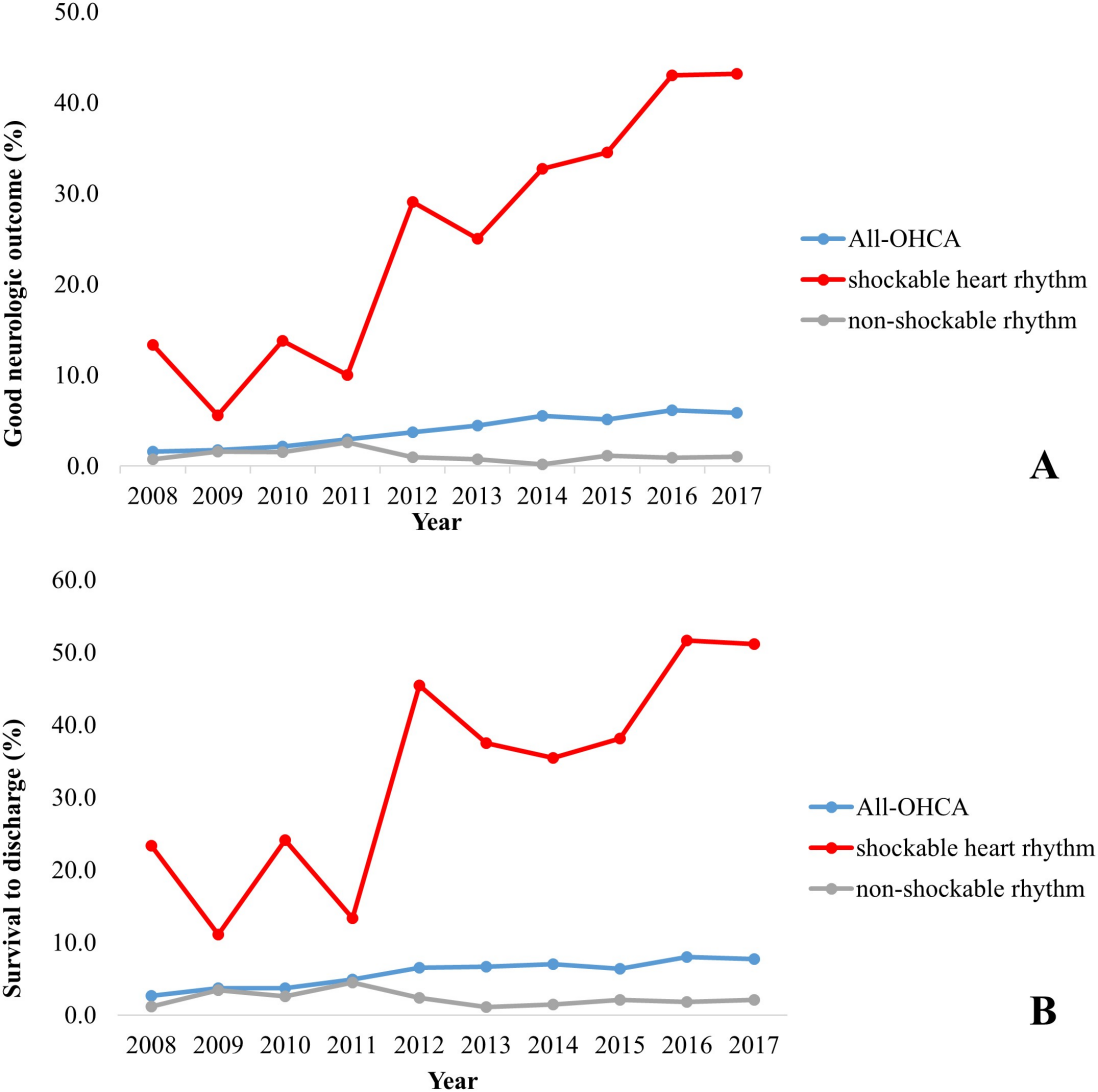
Trend of good neurologic outcome (A) and survival to discharge (B) according to primary ECG rhythm, Daegu, 2008–2017. OHCA, out-of-hospital cardiac arrest.

**Fig 3:**
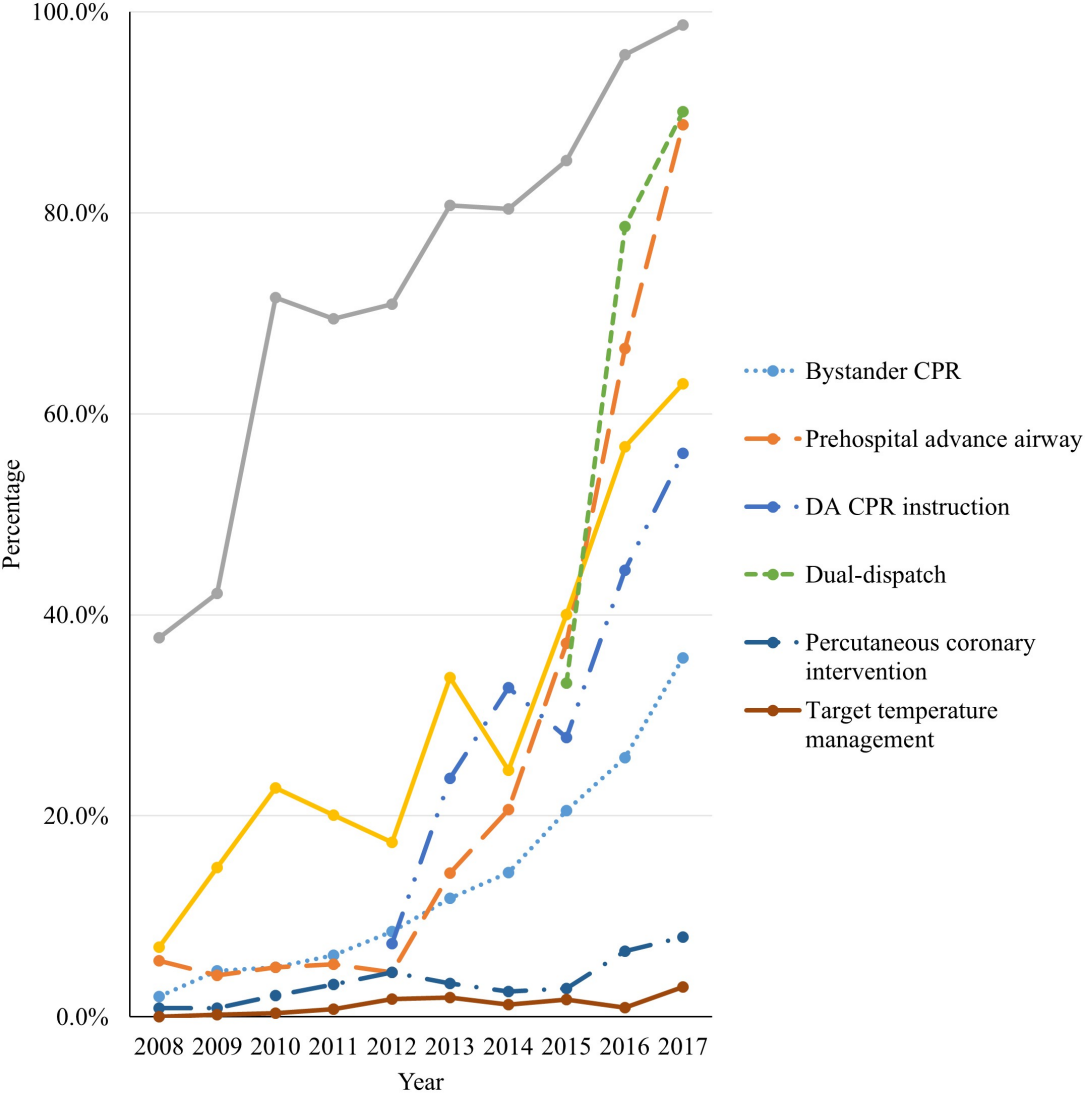
Trend in the interventions in patients with out-of-hospital cardiac arrest of medical origin in Daegu according to year. CPR, cardiopulmonary resuscitation; DA-CPR, dispatcher-assisted CPR.

The proportion of patients who survived with good neurologic outcomes increased from 1.5% in 2008 to 6.6% in 2017 (*P* for trend < 0.001). Among patients who had OHCA with an initial shockable rhythm, the proportion with good neurologic outcomes increased from 13.3% in 2008 to 46.0% in 2017 (*P* for trend < 0.001). The rate of survival to discharge increased from 2.6% in 2008 to 8.7% in 2017 (*P* for trend < 0.001). The rate of survival to discharge increased from 23.3% in 2008 to 55.0% in 2017 among patients with an initial shockable rhythm (*P* for trend < 0.001; Fig 2). Fig 3 shows trends in bystander CPR rate, DA-CPR instruction by dispatcher, and proportion of dual-dispatch performed for prehospital advanced airway management, for PCI, and for TTM by year, respectively. There were significant changes from 2008 to 2017 in bystander CPR (2.0% in 2008 vs 35.7% in 2017), DA-CPR instruction by dispatcher (7.3% in 2012 vs 56.1% in 2017), proportion of dual-dispatch (33.2% in 2015 vs 90.1% in 2017), performed for prehospital advanced airway management (5.6% in 2008 vs 90.1% in 2017), for PCI (0.9% in 2008 vs 8.0% in 2017), and for TTM (0.0% in 2008 vs 3.0% in 2017; *P* for trend <0.001).

### Outcome analysis

After adjustment for patient variables, the AORs (95% CIs) for good neurologic outcomes in model 2 were 1.20 (0.78–1.85) for phase 2 and 1.64 (1.09–2.46) for phase 3 compared with phase 1. The AORs (95% CIs) in model 2 were 1.10 (0.78–1.54) for phase 2 and 1.23 (0.89–1.71) for phase 3 for survival to discharge (Table 3).

**Table 3:** Multivariable logistic regression analysis for outcomes of study phase.

Group	Good CPC					Survival to discharge				
	No./ Total no.	%	AOR	95% CI		No./ Total no.	%	AOR	95% CI	
**Model 1**	259/6203	4.2				372/6203	6.0			
Phase 1	46/2124	2.2	Reference			82/2124	3.9	Reference		
Phase 2	86/1861	4.6	2.36	1.63	3.43	126/1861	6.8	1.88	1.40	2.52
Phase 3	127/2218	5.7	3.11	2.18	4.43	1164/2218	7.4	2.14	1.61	2.84
**Model 2**	259/6203	4.2				372/6203	6.0			
Phase 1	46/2124	2.2	Reference			82/2124	3.9	Reference		
Phase 2	86/1861	4.6	1.20	0.78	1.85	1236/1861	6.8	1.10	0.78	1.54
Phase 3	127/2218	5.7	1.64	1.09	2.46	1164/2218	7.4	1.23	0.89	1.71

AOR, adjusted odds ratio; CI, confidence interval; CPC, cerebral performance category.

Model 1: Adjusted for age, sex, and comorbidity (diabetes, heart disease, hypertension and stroke).

Model 2: Adjusted for age, sex, comorbidity (diabetes, heart disease, hypertension and stroke), location of arrest, primary electrocardiogram, witness status and response time interval.

For patients with a shockable rhythm, the AORs with 95% CIs in all adjusted models for phase 2 and phase 3 were significant for all outcomes compared with phase 1. The AORs (95% CIs) for good neurologic outcomes in model 2 were 3.76 (1.88–7.52) for phase 2 and 5.51 (2.77–10.98) for phase 3. For patients with non-shockable rhythm, the AORs (95% CIs) for good neurologic outcomes in model 2 were 0.47 (0.23–0.99) for phase 2 and 0.73 (0.41–1.33) for phase 3 (Table 4).

**Table 4:** Subgroup analysis for comparison of outcome by study phase according to primary ECG rhythm.

Group	Good CPC					Survival to discharge				
	No./ Total no.	%	AOR	95% CI		No./ Total no.	%	AOR	95% CI	
**Shockable rhythm**										
**Model 1**	195/633	30.8				245/633	38.7			
Phase 1	12/107	11.2	Reference			20/107	18.7	Reference		
Phase 2	76/261	29.1	3.05	1.54	6.01	100/261	38.3	2.41	1.37	4.24
Phase 3	107/265	40.4	4.99	2.54	9.81	125/265	47.2	3.40	1.93	5.99
**Model 2**	195/633	30.8				245/633	38.7			
Phase 1	12/107	11.2	Reference			20/107	18.7	Reference		
Phase 2	76/261	29.1	3.76	1.88	7.52	100/261	38.3	3.12	1.74	5.57
Phase 3	107/265	40.4	5.51	2.77	10.98	125/265	47.2	3.94	2.21	7.05
**Non-shockable rhythm**										
**Model 1**	64/5570	1.1				127/5570	2.3			
Phase 1	34/2017	1.7	Reference			62/2017	3.1	Reference		
Phase 2	10/1600	0.6	0.37	0.18	0.76	26/1600	1.6	0.51	0.32	0.82
Phase 3	20/1953	1.0	0.63	0.36	1.12	39/1953	2.0	0.65	0.43	0.98
**Model 2**	64/5570	1.1				127/5570	2.3			
Phase 1	34/2017	1.7	Reference			62/2017	3.1	Reference		
Phase 2	10/1600	0.6	0.47	0.23	0.99	26/1600	1.6	0.63	0.39	1.021
Phase 3	20/1953	1.0	0.73	0.41	1.33	39/1953	2.0	0.73	0.47	1.12

*Abbreviations*: ECG, electrocardiogram; AOR, adjusted odds ratio; CI, confidence interval; CPC, cerebral performance category.

Model 1: Adjusted for age, sex, and comorbidity (diabetes, heart disease, hypertension and stroke).

Model 2: Adjusted for age, sex, comorbidity (diabetes, heart disease, hypertension and stroke), location of arrest, witness status and response time interval.

## Discussion

This study demonstrated the effectiveness of our interventions to improve the emergency care system by using the Korean national population based OHCA registry. The study showed that the rate of bystander CPR increased substantially. The proportions of patients with survival to discharge and good neurologic outcome more than tripled during the 10-year study period in Daegu. Compared with phase 1, phase 2 and phase 3 showed significant improvements in good neurologic outcomes among patients with an initial shockable rhythm. The study showed the trend in survival and good neurologic outcomes after OHCA and the effect of community interventions during the 10-year study period. This study is meaningful because it analyzed the effectiveness of policy intervention to improve treatment performance for patients with OHCA over a decade in a single metropolitan city and aimed to identify the strong and weak part of regional emergency care system for patients with OHCA.

The trend toward increased survival after OHCA is likely multifactorial, including improvements in each of the links in the community, prehospital care during transport, and in-hospital treatment. Previous studies have reported that bystander CPR was associated with good neurologic outcomes after OHCA [10–12]. Implementation of DA-CPR increased bystander CPR rates, resulting in good neurologic outcomes after OHCA [10,13]. Resulting from community interventions, the rates of bystander CPR and DA-CPR instruction increased gradually, which may explain the increasing outcomes in Daegu.

During phase 3, the high-performance CPR program and the dual-dispatch system were implemented in the prehospital EMS, and feedback was provided to the communities, EMTs, and hospitals based on DEMSRe. The implementation of OHCA registry is the first action in the Utstein 10-step implementation strategy to improve OHCA outcomes [6,14], and it possibly contributed to the formation of cultural excellence for providing high-quality CPR by conducting regular feedbacks with prehospital EMTs. As a result of previous EMS interventions, the rates of dual-dispatch responses and advanced airway management gradually increased. The effect of the dual-dispatch system and advanced airway management on the improvement in outcomes after OHCA is unclear [7,15–17]. However, we expected positive effects by increasing the rapid success rate of prehospital advanced airway management and well-coordinated team approach in prehospital EMS. In phase 3, the prehospital epinephrine administration rate was only 3.5%. Previous studies reported that the survival to discharge was poorer as the time of the first epinephrine administration was delayed [18,19]. Although it was confirmed that the prehospital advanced care can be strengthened via prehospital EMS intervention, it is unfortunate that there were restrictions on the use of epinephrine due to the legal limitations of the scope of EMT’s practice. At the in-hospital stage, interventions to provide standardized post-cardiac arrest treatment were implemented, and accordingly, the rates of TTM and PCI also gradually increased and were associated with improved outcomes, as shown in Fig 3 and Supplementary Table 2.

Use of prehospital defibrillation by public AEDs in Daegu is still minimal compared with other countries [20,21]. The AED program for first responders, which is recommended in the 2015 AHA guidelines [3], has been implemented as a pilot program since 2017. This is expected to increase the use of public AEDs through a system linking the EMS dispatch center and nearest first responder. We found that the post-cardiac arrest treatment might be poor in Daegu. The use of treatments for post-cardiac arrest syndrome including PCI, TTM, and ECMO was lower than that in a previous multicenter study conducted in Korea [22]. The 2015 AHA guidelines and Korean guidelines for CPR recommend that comatose adults with return of spontaneous circulation after cardiac arrest have TTM regardless of the primary ECG rhythm [23,24]. Despite the possibility of selection bias, PCI was associated with good neurologic outcomes in previous studies [25,26]. Comparing 0.9%, 1.4% those with a non-shockable rhythm in phase 3, the rate of TTM and PCI with an initial shockable rhythm were much higher, i.e., 9.4% and 38.9%, respectively. Considering the rate of TTM and PCI performed, post-cardiac arrest treatment might contribute to different intervention effects according to primary ECG rhythm. To improve the outcomes of patients with non-shockable rhythm, post-cardiac arrest treatment such as TTM, which is also associated with good neurologic outcomes in patients with non-shockable rhythm [27], should be actively performed in hospital. In particular, early initiation of ECMO is associated with better neurologic and survival outcomes after OHCA [28,29], and only one patient received ECMO within 60 min after ED arrival in this study. Therefore, the late initiation of ECMO might have resulted in the poorer neurologic and survival outcomes (S1 Table). In future, intervention effort might be considered to change and actively perform treatments for post-cardiac arrest syndrome according to relevant guidelines and indications by conducting more educational programs for physicians and nurses at regional hospitals.

This study had a number of limitations that may restrict the generalizability of our results. First, this was not a randomized, controlled study. The possibility of potential biases could have affected our results. Second, the competence levels and experience of prehospital EMTs, in-hospital CPR performance levels, and clinical and physiological characteristics in the post-resuscitation state were not considered; such variables may have influenced our results. Third, there were significant changes in CPR guidelines and post-cardiac arrest treatment during the study period. Although the effects of intervention related factors were shown in the S1 Table, there might be still limitations in analyzing the effects of changes in care for cardiac arrest and the post cardiac arrest syndrome. Fourth, because a difference in the proportion of shockable rhythm among phase groups exists, a difference in the proportion of patients subject to PCI and TTM might also be noted. This may have been a selection bias, affecting the outcome due to differences between performing PCI and performing TTM. Fifth, this study was conducted in an EMS system at an EMT-intermediate level, and there may be differences in emergency care systems for each country. Therefore, the study findings should be only cautiously generalized.

In this study, the community, local government officers, prehospital EMSs, fire departments, and Daegu Emergency Medical Collaboration Committee worked and cooperated together to improve the chain of survival and emergency care system. We found citywide improvement in good neurologic outcomes in patients who experienced OHCA, especially in those with initial shockable rhythm.

## Supporting information

S1 Table Multivariable logistic regression analysis for outcomes according to factors related intervention. (DOC)
