# Supplementary material for: Effect of citywide enhancement of the chain of survival on good neurologic outcomes after out-of-hospital cardiac arrest from 2008 to 2017
Source: PLoS One. 2020 Nov 6;15(11):e0241804. doi: 10.1371/journal.pone.0241804 (PMC7647071; doi:10.1371/journal.pone.0241804)
Supplement: S1 Table — (DOC) [file pone.0241804.s001.doc]

~~S1 Table. Multivariable logistic regression analysis for outcomes according to factors related intervention~~

| **Factors related intervention** | **Good CPC** | | | **Survival to discharge** | | |
| --- | --- | --- | --- | --- | --- | --- |
|  | **AOR^a^** | **95% CI** | | **AOR^a^** | **95% CI** | |
| **Bystander CPR** | 1.70 | 1.12 | 2.56 | 1.73 | 1.20 | 2.49 |
| **Dual dispatch vs single dispatch** | 0.87 | 0.53 | 1.42 | 1.02 | 0.65 | 1.59 |
| **Prehospital advance airway** | 0.83 | 0.54 | 1.29 | 1.01 | 0.69 | 1.47 |
| **Percutaneous coronary intervention** | 16.84 | 10.64 | 26.65 | 23.63 | 14.91 | 37.46 |
| **Target temperature management** | 4.09 | 1.95 | 8.59 | 9.43 | 4.64 | 19.17 |
| **Extracorporeal membrane oxygenation** | 0.09 | 0.03 | 0.28 | 0.07 | 0.02 | 0.21 |

*Abbreviations:* AOR, adjusted odds ratio; CI, confidence interval; CPC, cerebral performance category; CPR, cardiopulmonary resuscitation; EMT, emergency medical technician

^a^Adjusted for age, gender, comorbidities (diabetes, heart disease, hypertension and stroke), location of arrest, primary electrocardiogram (ECG), witness status, response time interval, scene time interval, transport time interval, and level of emergency department.
